# Recipes for Inducing Cold Denaturation in an Otherwise Stable Protein

**DOI:** 10.1021/jacs.1c13355

**Published:** 2022-04-15

**Authors:** Angela Bitonti, Rita Puglisi, Massimiliano Meli, Stephen R. Martin, Giorgio Colombo, Piero Andrea Temussi, Annalisa Pastore

**Affiliations:** † Department of Molecular Medicine, University of Pavia, Via C Forlanini 6, 27100 Pavia, Italy; ‡ UK Dementia Research Institute at the Maurice Wohl Institute of King’s College London, London SE5 9RT, United Kingdom; § Istituto di Scienze e Tecnologie Chimiche “Giulio Natta” (SCITEC), CNR, Via Mario Bianco 9, 20131 Milano, Italy; ∥ Structural Biology Technology Platform, The Francis Crick Institute, 1 Midland Rd, London NW1 1AT, United Kingdom; ⊥ Department of Chemistry, University of Pavia, Via Torquato Taramelli, 12, Pavia 27100, Italy

## Abstract

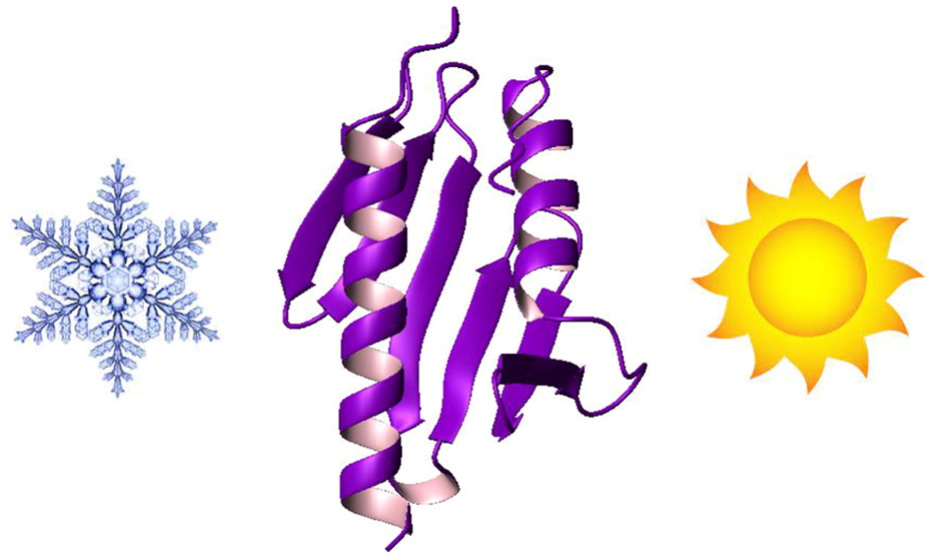

Although cold denaturation is a fundamental phenomenon common to all proteins, it can only be observed in a handful of cases where it occurs at temperatures above the freezing point of water. Understanding the mechanisms that determine cold denaturation and the rules that permit its observation is an important challenge. A way to approach them is to be able to induce cold denaturation in an otherwise stable protein by means of mutations. Here, we studied CyaY, a relatively stable bacterial protein with no detectable cold denaturation and a high melting temperature of 54 °C. We have characterized for years the yeast orthologue of CyaY, Yfh1, a protein that undergoes cold and heat denaturation at 5 and 35 °C, respectively. We demonstrate that, by transferring to CyaY the lessons learnt from Yfh1, we can induce cold denaturation by introducing a restricted number of carefully designed mutations aimed at destabilizing the overall fold and inducing electrostatic frustration. We used molecular dynamics simulations to rationalize our findings and demonstrate the individual effects observed experimentally with the various mutants. Our results constitute the first example of rationally designed cold denaturation and demonstrate the importance of electrostatic frustration on the mechanism of cold denaturation.

## Introduction

Understanding the molecular forces that induce proteins to fold/unfold and determine their thermodynamic stability is a major challenge of physical chemistry. Studies of how proteins preserve their fold as a function of environmental factors such as temperature, pH, pressure, or solvent composition have been a key tool in helping in this endeavor. While it is well known that proteins unfold at high temperature, only relatively recently it has been possible to gain information on how proteins in aqueous solutions unfold also at low temperature and undergo cold denaturation. Discovered in the last century,^1−3^ this transition is thought to be caused by the attenuation of hydrophobic forces and the strengthening of hydrogen bonds to the solvent at low temperature, resulting in the opening of the structure.^4^ It has been theoretically demonstrated, based on fundamental thermodynamic considerations, that all proteins would be seen to undergo cold denaturation if they could be studied at sufficiently low temperatures.^5^ However, it is impossible to observe cold denaturation for the majority of proteins because it occurs at temperatures well below the freezing point of water. For this reason, the cold transition has been rarely studied and only recently has its mechanism started to be revealed.^6^

A key role in the understanding of the cold denaturation process has been played by studies on Yfh1, a marginally stable protein that, in the absence of salts, undergoes cold denaturation at temperatures around 5 °C and has a high-temperature unfolding transition at around 35 °C.^7^ This is an unusual protein. There are other examples of proteins able to undergo cold denaturation under *ad hoc* conditions as, for instance, the introduction of destabilizing mutations,^8,9^ external agents like pressure,^10^ the presence of extraordinary environments like reverse micelles,^11^ or the addition of a denaturant.^12^ Nevertheless, Yfh1 is probably the only example of a natural protein with an observable cold denaturation under (quasi) physiological conditions. Because of its properties, Yfh1 has been used extensively as an ideal tool for exploring protein stability under a wide variety of environmental conditions.^13−15^ Yfh1 is the yeast orthologue of frataxin, a protein highly conserved from bacteria to primates that plays a crucial role in the human neurodegenerative disease Friedreich ataxia.^16^ The structure of this protein is a mixed αβ-fold in which two N- and C-terminal helices pack against a 5–7-strand antiparallel β-sheet, depending on the orthologue.^17^ Although sharing the same fold, frataxins from different species have quite different fold stabilities, with the yeast orthologue so far being the most unstable. CyaY, the *Escherichia coli* orthologue, for instance, does not undergo cold denaturation at detectable temperatures and has a high-temperature unfolding transition at around 54 °C.^18^

Protein stability is the resultant of several different stabilizing and destabilizing forces.^19−21^ One of the factors that determine the stability of the frataxin family was identified in a C-terminal extension of variable length among the orthologues. This structural element folds back and packs against a groove formed by helices 1 and 2 (α1 and α2), protecting the hydrophobic core (for a review on the frataxin structure, see Pandolfo and Pastore^22^). The length of this extension seems to correlate with the stabilities of the various orthologues: the C-terminal tail is, for instance, short in Yfh1, but up to three and eight residues in the bacterial and human orthologues, respectively.^18,23^ Accordingly, we demonstrated that the C-terminal truncation of CyaY leads to a significant reduction of the melting temperature of the protein and thus to a reduction of its overall stability.^18^ It was also independently proposed that, at a molecular level, cold denaturation of Yfh1 could be facilitated by the presence on the surface of the protein of a destabilizing hot spot that allows the entrance of water molecules into the hydrophobic core at low temperature.^6,15^ This hot spot in Yfh1, named “electrostatic hinge”, is characterized by the spatial proximity of four negatively charged residues, E89, E103, D101, and E112, in the secondary elements α1, β1, β1, and β2, respectively. In a previous study, we demonstrated that it is possible to eliminate cold denaturation and stabilize Yfh1 by up to 14 °C, simply by mutating just one of the acidic residues of the hinge to a neutral serine.^6^ Based on this evidence, we hypothesized that repulsion among these negatively charged amino acids could create a spot of electrostatic frustration and make the hydrophobic core of the protein more accessible to the solvent, in agreement with the mechanism of solvation of core residues that occurs during cold denaturation according to Privalov’s model.^4^ This model proposes that, at low temperature, the interactions of proteins with the solvent become dominant while the hydrophobic forces are less important. This results in the opening of the hydrophobic core and unfolding.

In the present work, we reasoned that if we could convert the much more stable CyaY orthologue, which does not undergo cold denaturation, into a protein that is not only less stable but also that undergoes cold denaturation, we would prove our understanding of the mechanisms governing the process. We thus designed a number of CyaY mutants acting both on the C-terminus to destabilize CyaY to obtain a heat denaturation point comparable to Yfh1 and on introducing an electrostatic hinge to understand how these insults would affect protein stability. We demonstrate that just a few such carefully designed mutations are able to induce cold denaturation. As a control, truncation of the protein together with stabilization through other interactions leads to a marginally stable protein but not to cold denaturation. Our results provide a clear rationale for introducing cold denaturation in proteins and shed new light on the subtle equilibrium, which is selected out through an evolutionary process between protein stability and survival to allow protein turn-over and functionality.

## Results

### Structure Analysis Informs Mutant Design

Our goal was to induce the same behavior of Yfh1 in the other much more stable CyaY. This is a protein of 106 residues that has a melting temperature (*T*_m_) for heat unfolding of 54 °C and no detectable cold denaturation. We reasoned that we should first make the protein marginally stable because otherwise the electrostatic frustration would be easily neutralized by other stabilizing forces. We therefore cut the C-terminus by three residues, truncating the protein at a position that corresponds to the length of Yfh1. We had previously demonstrated that this choice results in a destabilized CyaY(1-103) mutant (hereafter named CyaY^103^) with a high-temperature melting point comparable to that of Yfh1.^18^ Since, however, we had not yet discovered the cold denaturation transition of Yfh1, we had not explored the behavior of CyaY^103^ at low temperatures.

**Figure 1:**
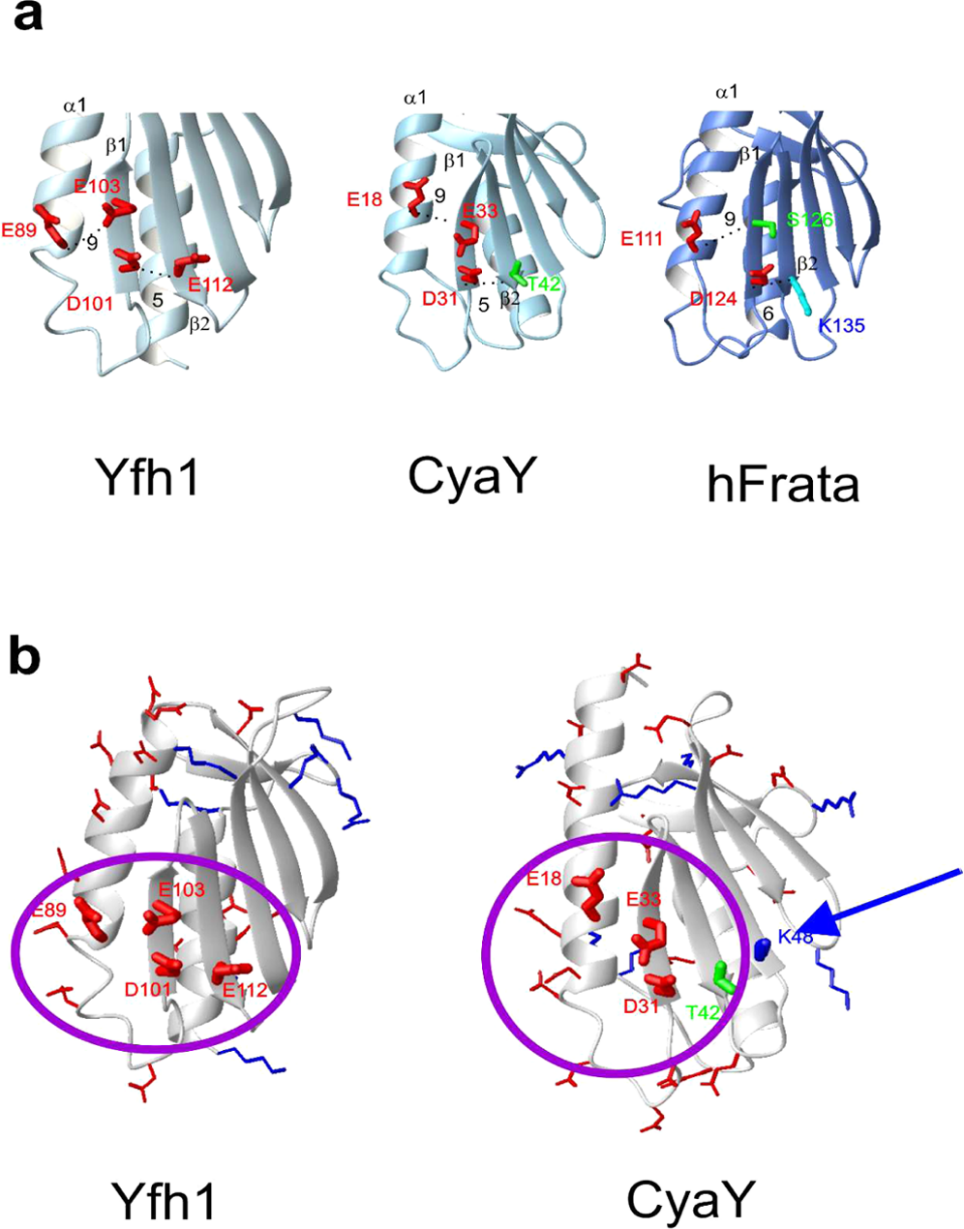
Comparison of the hinge regions of frataxins. (A) Ribbon structures of Yfh1, CyaY, and human frataxin showing the residues of the electrostatic hinge observed in Yfh1 and the corresponding residues in CyaY and human frataxin. (B) Close-up of the regions of Yfh1 and CyaY showing the potentially compensatory role of K48.

We then noticed that there is no electrostatic hinge in CyaY. In Yfh1, the hinge is formed by the four acidic residues E89, D101, E103, and E112 (Figure 1). The corresponding residues in CyaY are E18, D31, E33, and T42. For comparison, the much more stable human frataxin has E111, D124, S126, and K135 in the corresponding positions.^18^ Starting from the CyaY^103^ mutant, we first considered a T42E mutant as a way of introducing the hinge in this protein. However, we also noticed that the repulsion introduced by the T42E mutation could be at least partially compensated for by the presence, on an adjacent β-strand, of K48. We thus hypothesized that it would be insufficient to only mutate T42 to E and decided to mutate also K48 to E. We produced two mutants: CyaY^103^T42E_K48E (hereafter dubbed for simplicity EE) in which we substituted the positively charged amino acid with a negatively charged one and, as a control, CyaY^103^T42E_K48T (ET) that introduced a milder polar amino acid at the same position. Finally, we noticed that the nearby T40 is also in an excellent position to create the negatively charged quadrilateral structure of the hinge. Therefore, we designed a mutant CyaY^103^T40E_T42E_K48E (EEE) and its milder version CyaY^103^T40E_T42E_K48T (EET) in which additional negative charges were added in the same area.

All proteins were cloned and expressed in *E. coli*. Despite several attempts, however, we were unable to obtain EEE, which persistently expressed in inclusion bodies even changing the induction temperature (data not shown). The other four proteins could instead be easily expressed in their soluble forms and could be purified to the necessary levels. This difference suggests that the mutations affect the variants differently and that the presence of a negatively charged surface may interfere with proper folding of the proteins.

### Circular Dichroism (CD) Spectra of the Mutants Reveal Different Degrees of Structural Content at Different Temperatures

**Figure 2:**
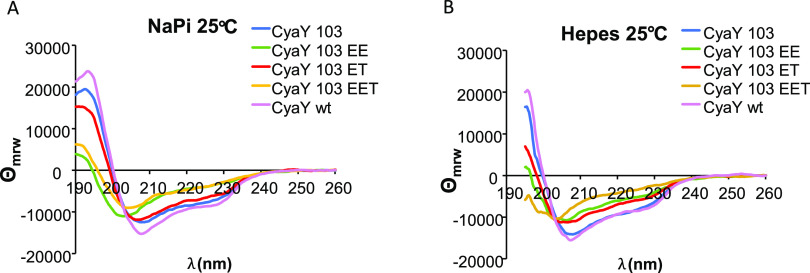
CD spectra of CyaY and its mutants at 25 °C. CyaY is shown in magenta, CyaY^103^ is shown in blue, EE is shown in green, ET is shown in red, and EET is shown in yellow. The samples were in (A) 20 mM sodium phosphate (NaPi) pH 7.4 and (B) 10 mM Hepes pH 7.

We first evaluated the effects of the mutations on the CyaY-fold by monitoring the secondary structures of the mutants by CD spectroscopy. The spectrum at room temperature of the wild-type protein was compared to that of the truncated form using two different buffers, *N*-(2-hydroxyethyl)piperazine-*N*′-ethanesulfonic acid (Hepes) and sodium phosphate. It is important to recall that cold denaturation was only observed in the yeast protein, Yfh1, when the salt content was minimal. From this point of view, it is worth noticing that Hepes, also in the present study, was always used without any added salt, whereas phosphate buffer is inherently a “salty” buffer. In both buffers, the spectrum of wild-type CyaY at 25 °C had all of the features expected for a folded protein with an appreciable content of helical structure (ca. 30%), in agreement with the three-dimensional structure.^17,24^ The spectrum of CyaY^103^ was somewhat less intense than that of the full-length protein, indicating a small loss of secondary structure. In Hepes, the spectra of CyaY^103^, EE, and ET were similar (Figure 2A). The spectrum of EET revealed instead a shift of the minimum from 205–208 to 203 nm and a weakening of the band at 222 nm, indicating further loss of structure. The spectra of the same proteins in phosphate at the same temperature are qualitatively similar, except that in this case, the spectra of both EE and EET have a similar shift toward lower wavelengths and an appreciable weakening of the band at 220 nm (Figure 2B).

These results suggest a different degree of stability of the various variants already at room temperature.

### Introducing an Electrostatic Hinge Affects the CyaY Stability

**Figure 3:**
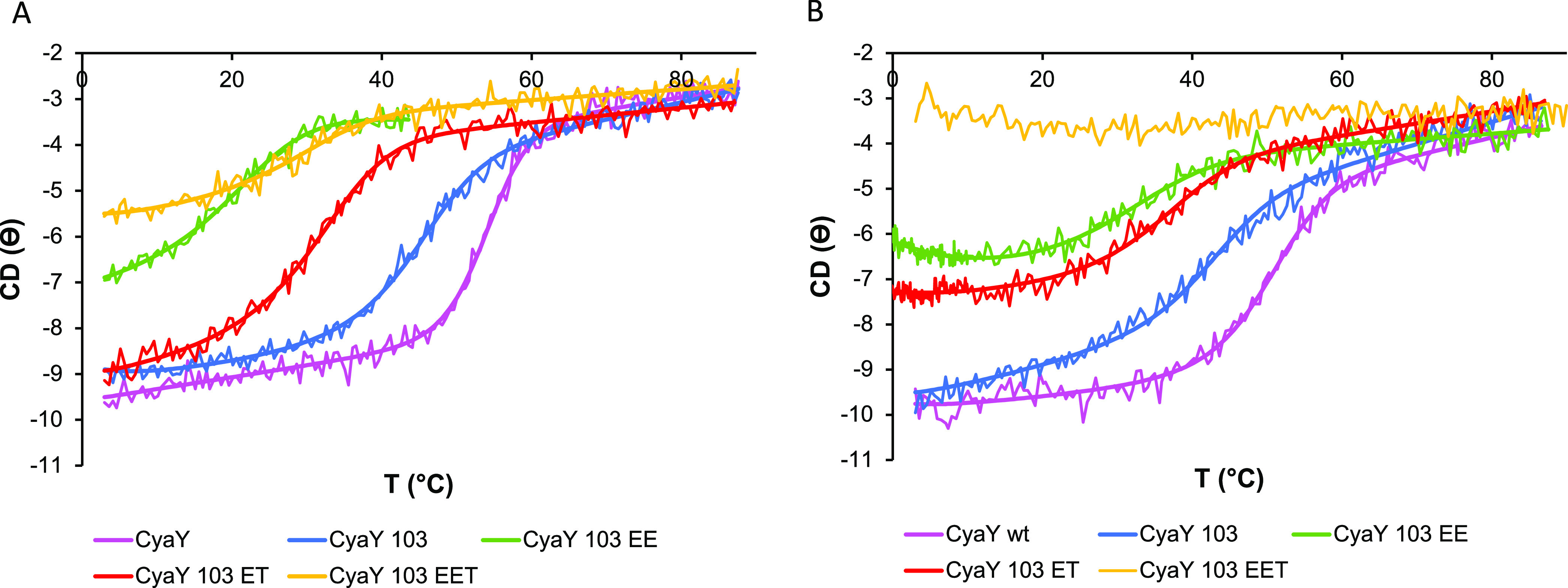
Thermal denaturation spectra of CyaY and its mutants. CD spectra as a function of temperature following the wavelength at 222 nm in (A) 20 mM NaPi at pH 7.4 and (B) 10 mM Hepes at pH 7. The color coding is indicated below the figure. The curves in Hepes of CyaY^103^ and the ET and EE mutants are the resultants of merging data collected, with a scanning rate of 1 °C/min and data collected between −1 and 10 °C at 0.2 °C/min and with a long time constant (8 s) to reduce noise. We offset the data to match the value at 10 °C.

We then proceeded to study the temperature dependence of the fold to quantify thermal stability. We compared the CD melting curves of the proteins by monitoring the ellipticity at 222 nm in the temperature range of 3–90 °C. The choice of this wavelength was dictated both by the consideration that it is extremely sensitive to even small changes in the secondary structure and because it is intrinsically less affected by potential sources of noise usually observed at lower wavelengths. The curves were markedly different with a different behavior in Hepes and phosphate and with a relatively higher cooperativity in phosphate (Figures 3 and S1, Supporting Information). All transitions were fully reversible, as previously reported.^18^ The curves showed progressive destabilization: wild-type CyaY is the more stable variant, with *T*_m_ values of 54 and 50 °C in phosphate and Hepes buffers, respectively. The values of wild-type CyaY and CyaY^103^ are in full agreement with those previously reported (Table 1).^18^ Among the mutant constructs, ET has an intermediate stability, with *T*_m_ values of 35 °C in both buffers but in Hepes, the low-temperature pretransition is flatter, suggesting that the protein could tend toward a low-temperature transition. EE is further destabilized and has a melting point of 33 °C in phosphate. In Hepes, it has a high-temperature transition at ca. 35 °C but also a clear tendency toward a second transition at low temperature. To further substantiate the presence of cold denaturation, we measured again the curve of unfolding of CyaY^103^and the ET and EE mutants, starting from low temperature (−1 °C) up to 10 °C, at a slow scanning rate (0.2 °C/min as compared to 1 °C/min used in the previous scans) and with a long time constant (8 s) to reduce noise. The signals obtained were slightly shifted as compared to the previous curves since with single wavelength scans there is no baseline correction. We thus offset the data to match the value at 10 °C. The resulting curves confirmed that the pretransition behavior of both the EE and, to a minor extent, the ET mutant is consistent with the presence of a cold transition (Figure 3). Finally, the triple mutant EET has a *T*_m_ of 29 °C in phosphate and no measurable temperature dependence in Hepes, in which the curve is substantially flat. This implies that the protein is unfolded at all temperatures and does not thus have any temperature dependence.

**Table 1:** Summary of the Thermodynamics Parameters of CyaY and Its Mutants. For most of the proteins, the value of Δ*C*_p_ was fixed to 1.5 kcal/(mol K)

	Δ*H(kcal/mol)*	*T*_m_ (°C)	Δ*C*_p_ (kcal/(mol K))
CyaY			
Hepes	50.2 ± 2.5	50.0 ± 0.9	fixed
NaPi	77.1 ± 3.9	53.8 ± 0.2	fixed
CyaY^103^			
Hepes	42.1 ± 3.1	41.6 ± 0.5	fixed
NaPi	44.1 ± 3.2	44.1 ± 0.3	fixed
EE			
Hepes	18.4 ± 2.2	26.5 ± 1.5	1.21 ± 0.15
NaPi	33.3 ± 4.8	21.7 ± 2.4	fixed
ET			
Hepes	34.1 ± 1.9	36.6 ± 1.3	fixed
NaPi	38.2 ± 2.9	31.7 ± 0.3	fixed
EET			
Hepes			
NaPi	29.9 ± 4.1	28.5 ± 1.2	fixed

We can thus conclude that the introduction of electrostatic frustration in a destabilized version of CyaY is able to not only influence the high-temperature transition but also induce cold denaturation.

### Stability Curves and the Thermodynamic Parameters

To quantify the results, we extracted the thermodynamic parameters from the data (Table 1). We assumed that unfolding transitions are, as a first approximation, two-state processes from folded (F) to unfolded (U) states. We postulated that the Δ*C*_p_ values of the two forms do not depend on temperature. When these two conditions are reasonably well met, the populations of the two states at temperature *T*, *f*_F_(*T*) and *f*_U_(*T*), are a function of the Gibbs free energy of unfolding, Δ*G*°(*T*).^14^ In this case, it is possible to extract the heat melting temperature, *T*_m_, and the enthalpy difference at the melting point, Δ*H*_m_, using the thermal dependence of measurements such as CD spectra also in the absence of a reliable estimate of the heat capacity difference at constant pressure (Δ*C*_p_). The fitting of these data is in fact completely insensitive to the value of Δ*C*_p_. Conversely, when cold denaturation is observable above the freezing point of water, it is in principle possible to extract all thermodynamic parameters for the unfolding process from the thermal dependence of the CD signal,^25^ assuming that previous conditions are met.^4,14^ Variations in the CD signal directly depend on fractions of folded and unfolded proteins present, and the thermodynamic parameters, *i.e.*, *T*_m_, Δ*H*_m_, can be determined from the unfolding curves by fitting the CD signal between −1 and 90 °C with a nonlinear fit (damped least-squares method, also known as the Levenberg–Marquardt algorithm).^26,27^ From these parameters, it is possible to obtain the dependence of the free energy of unfolding as a function of temperature, also referred to as the stability curve of a protein.^5^ Other parameters, *e.g*., the low-temperature unfolding (*T*_c_), can be read from the stability curve.

In the case of the CyaY constructs reported in the present study, only two of the mutants show signs of cold denaturation, whereas the data for CyaY itself are typical of a stable globular protein that shows only heat unfolding at temperatures higher than room temperature. The transition at low temperature of ET is, however, only at the very beginning in the range of temperatures investigated, and we found it difficult to obtain all of the thermodynamic parameters. Thus, for all of the variants except EE, we imposed a fixed value of Δ*C*_p_ consistent with values predicted for proteins of this size.^28^ Conversely, the data for the EE mutant were sufficiently good for a reliable determination of Δ*C*_p_ from data fitting, which resulted in a value of 1.21 kcal/(mol K). This value is in agreement with what we had previously obtained for Yfh1, which has the same structure as CyaY and presumably similar accessible surface area given the similarity of the unfolding behavior.^14^

**Figure 4:**
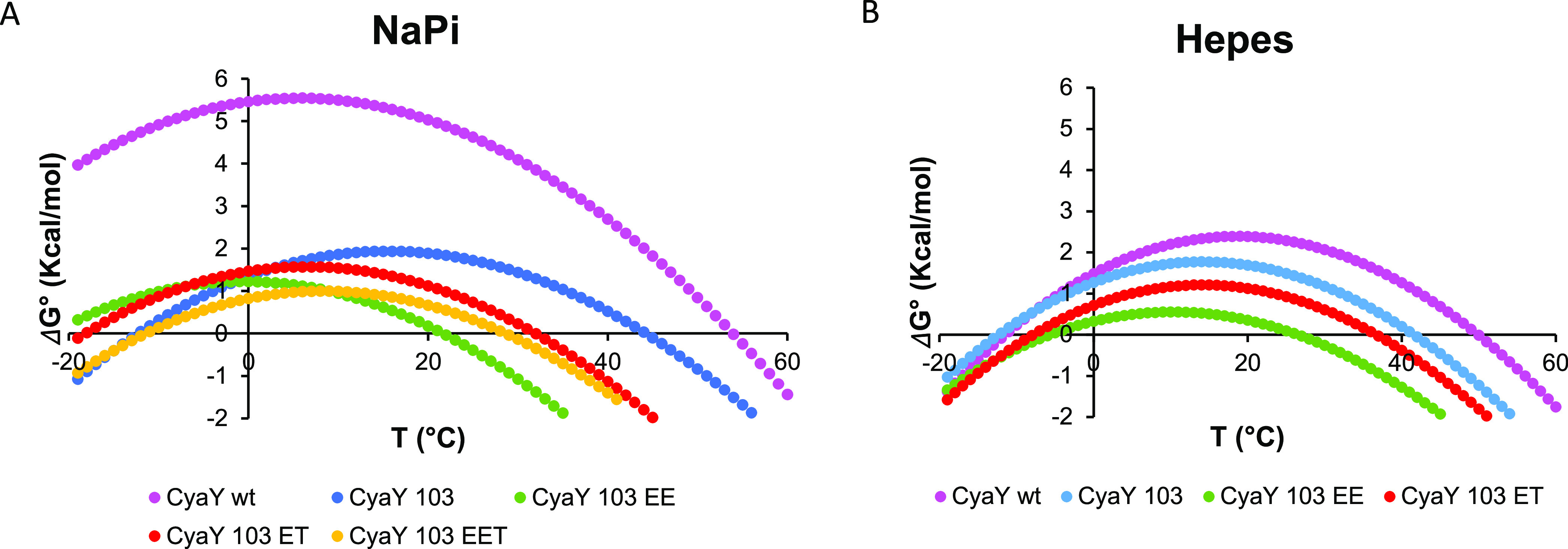
Stability curves as obtained from the Gibbs–Helmholtz equation with thermal parameter from fitting for CyaY and its mutants in (A) 20 mM NaPi at pH 7.4 and (B) 10 mM Hepes at pH 7. The *y*-axis refers to the standard state unfolding free energy. No stability curve is reported for EET in Hepes since the protein remains unfolded in the whole temperature range. The color coding is the same as in Figure 3.

The main aspect in a comparison of the stability curves obtained is the difference between a generic destabilization and mutations addressed to the specific spot on the protein surface that contains the electrostatic hinge (Figure 4). If, for instance, we compare the stability curves of wild-type CyaY and CyaY^103^, it is clear that the main consequence of the truncation is an overall downward shift of the curve, corresponding to a decrease in Δ*H*, with a maximum stability temperature (*T*_s_) comparable for the two curves. Mutations specifically addressed to make the electrostatic hinge similar to that of Yhf1 yielded curves with a more complex behavior, especially in Hepes: in the case of the EE mutant, it is possible to observe clearly the onset of cold denaturation also in the stability curve because the low point of the curve is close to water freezing. The effect is less pronounced with ET. This means that we can differentially introduce a generic destabilization or cold denaturation by rationally playing on the stabilizing/destabilizing forces of a protein.

### CyaY C-Terminus as the Doorkeeper That Prevents Water from Entering into the Hydrophobic Core

**Figure 5:**
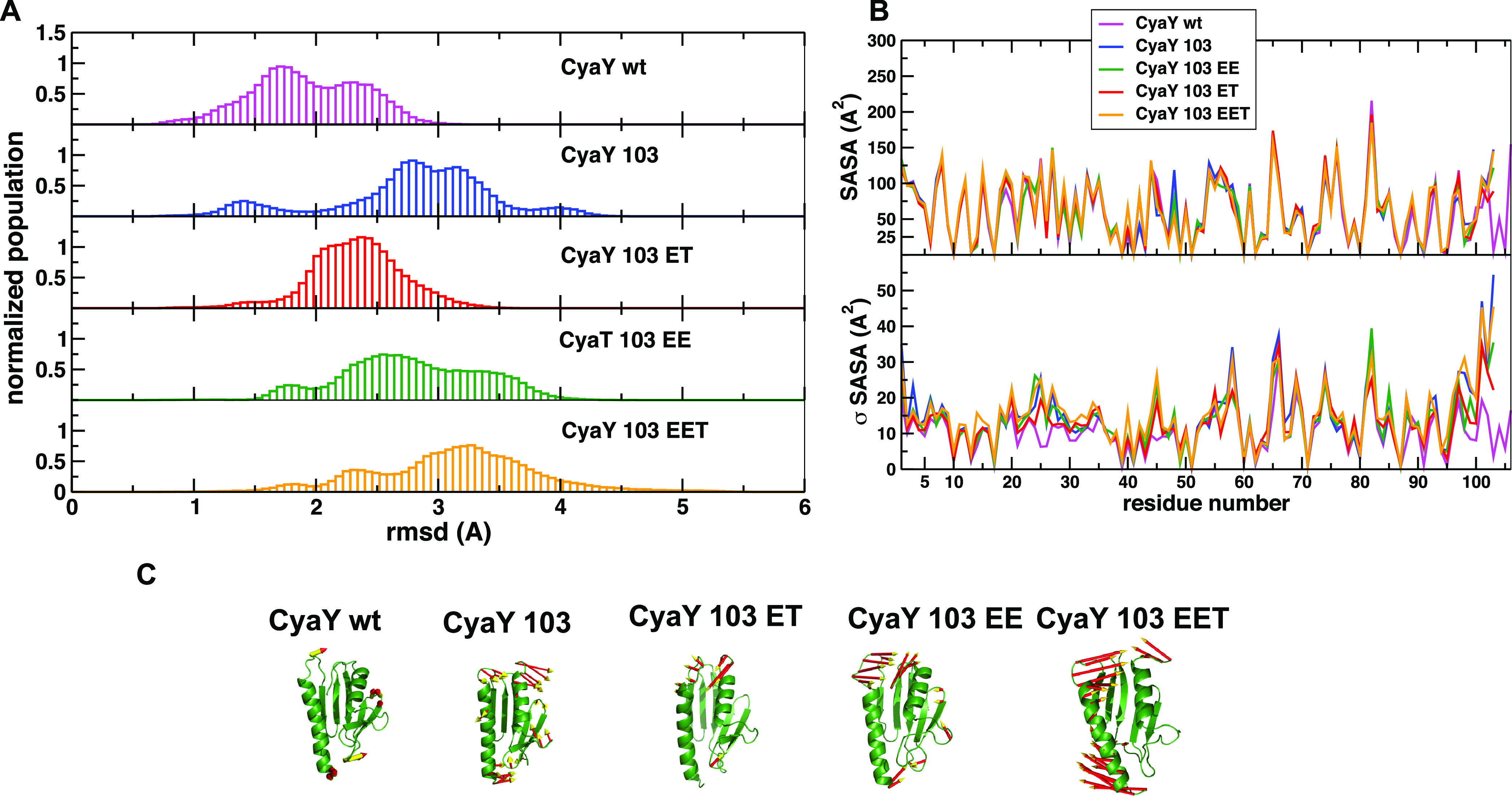
MD characterization of CyaY structural dynamics. (A) Distributions of the root-mean-square deviations (RMSDs) from the reference native structure along the equilibrated parts of the trajectories. (B) Average and standard deviations of the per-residue accessible surface area (SASA) for the simulation of each mutant. (C) Principal component analysis (PCA) analysis: the principal motions of each mutant are evidenced for each trajectory by projecting the respective simulation on the first eigenvector obtained from the principal component analysis of the simulation.

To get a visual mechanistic picture of the effects of our mutations, we ran molecular dynamics (MD) simulations. It is unrealistic to think of following the whole unfolding process observed in thermal unfolding with the current sampling methods,^29^ but we reasoned that we could nevertheless get valuable indications by comparing the breathing motions and the internal dynamics of the different variants as compared to the wild-type native state. Analysis of the distribution of the root-mean-square deviations (RMSDs) from the reference native structure along equilibrated parts of the trajectories showed that the values for WT CyaY are consistently lower than for the mutants (Figure 5A). Interestingly, the mutant with the RMSD distribution peak located at higher values is EET. Deletion of the C-terminal S104, F105, and R106 in CyaY^103^ and the other mutants breaks the interactions of the C-terminus with the core of the protein, especially the hydrophobic and π interactions involving F105 with R20 and Trp24. The net effect is an opening motion of the C-terminus in all of the mutants, whose extent correlates with the progressive loss of stability of the corresponding proteins.

Next, we evaluated the average and standard deviations of the per-residue accessible surface area (SASA) for each simulation (Figure 5B). While the average values are overall similar, an exception can be noticed in the mutant C-termini, in which we observed a clear increase in solvent accessibility for EE. The presence of a difference between variants tells us that we are seeing the combined effect of the shortening and of the electrostatic strain. Most interestingly, the deviations from the mean (standard deviation) highlight three areas where a significant breathing of the protein may take place, modifying the potential accessibility to the solvent. These regions comprise residues 3, 6, 23 to 29, 45, and the C-termini. Such modulations in response to the solvent must be reconnected to a general modification of the breathing motions of the mutants as compared to that of the wild type.

We further carried out for each system principal component analysis (PCA) on the covariance matrices obtained from the combined trajectories. Essential dynamics reduces the dimensionality of the covariance matrix by diagonalization. This method describes the global protein motions that are represented by the matrix eigenvectors and eigenvalues of the respective covariance matrices. Essential dynamics emphasizes the amplitude and direction of dominant protein motions. Since the magnitudes of displacements along the main eigenvectors are represented by their eigenvalues, it is possible to evidence the principal components of the protein global motions by sorting them after calculating the main eigenvector for each trajectory and projecting the respective simulation on it. While no major displacement was observed for the wild type, the loop motions between α1 and β1 (that is close to the C-terminus in the wild type) became prominent for all mutants (Figure 5C). The open-to-close motions of this loop could expose the core of the protein to solvent penetration, an effect that might be combined with that caused by removal of the C-terminus.

**Figure 6:**
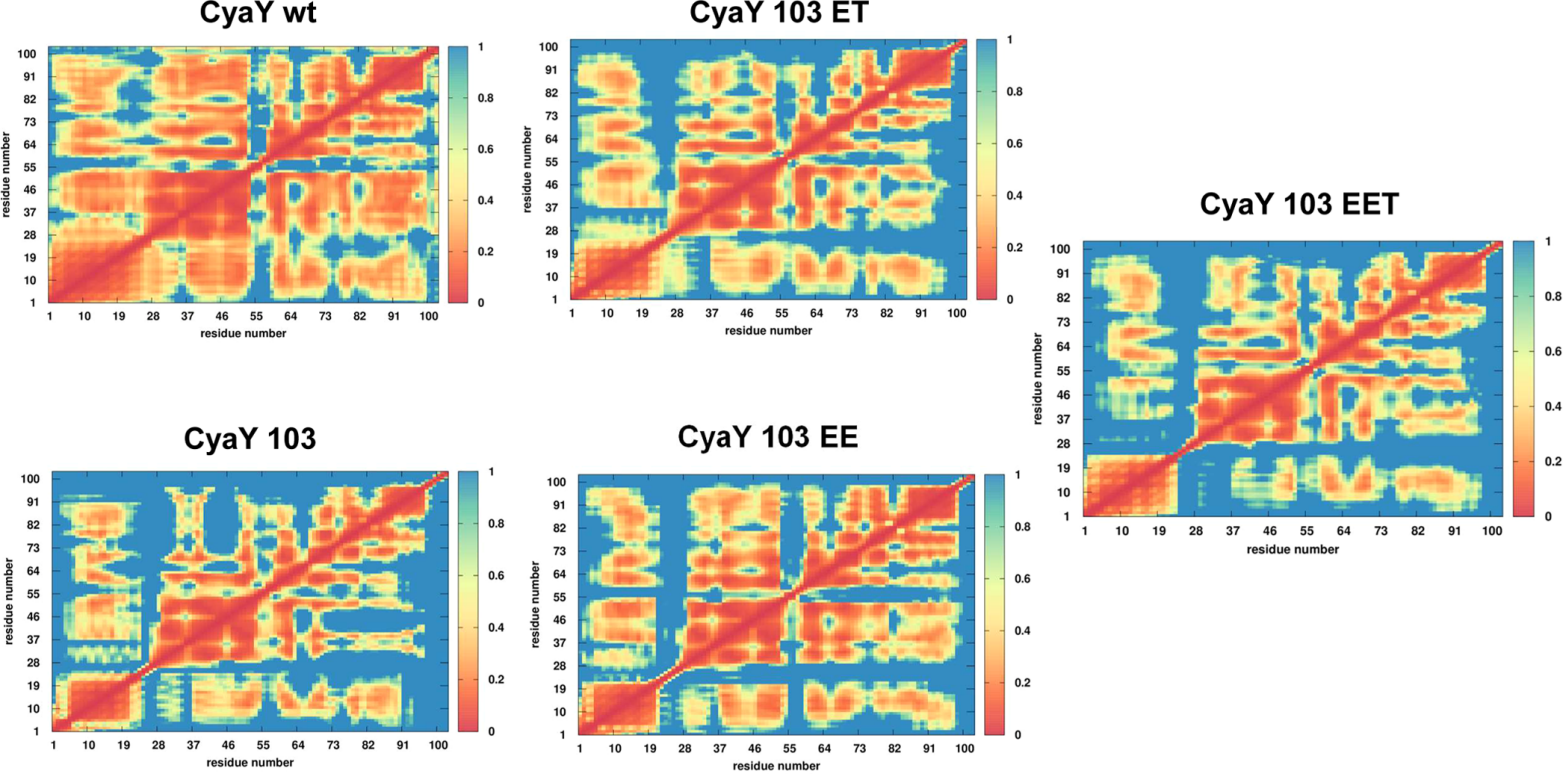
Matrices depicting the PDF values for each of the mutants reported in this study.

Finally, we computed the fluctuations of pairwise amino acid distances. Pairwise distance fluctuations (PDFs) allow the measurement of the degree of internal coordination of residue pairs in a given structure (Figure 6). The lower the PDF values, the lower the fluctuations and the higher the internal coordination in the structural ensemble are. Consequently, this parameter reports on the tendency of a protein to fluctuate around or to diffuse out of a structural ensemble. The more internally connected a certain structure, the more it will tend to stay in a specific conformational ensemble. In this case, the wild-type protein appeared to be more internally connected/coordinated than all other mutants. In other words, residue pairs throughout the three-dimensional (3D) fold tend to fluctuate around the distances that are typical of the wild-type fold. In contrast, the mutants feature substantially higher values of the fluctuations, indicating that the protein breathing motions are much larger, prompting the molecule to visit alternative conformational states. This observation could be reconnected to the fact that the wild type has a pronouncedly lower tendency to leave the native state as compared to the mutants.

Taken together, these results provide a clear picture of the events that result in cold denaturation: the transition is induced by the destabilization introduced by the protein truncation, which leads to the lack of a fold sealer that protects the hydrophobic core. When additional destabilization is introduced by electrostatic frustration, the strain introduced by the latter repulsion leads to an increase of the overall motions and allows opening of the overall fold. When this effect becomes so extreme as in EET (and EEE), the protein becomes so unstable that it cannot fold and either goes into inclusion bodies or can be purified but unfolds during purification.

## Discussion

Understanding the molecular bases of protein stability remains a key aspect in biophysics and protein folding. Within this topic, cold denaturation has offered a unique tool of investigation because, through the possibility of determining the complete stability curve, it allows assessment of all of the thermodynamics parameters, which could otherwise be out of reach. Unfortunately, the number of proteins that undergo cold denaturation under detectable conditions is limited. For many years, we used Yfh1, the yeast orthologue of frataxin, to study the determinants of cold denaturation in this protein, which is a uniquely convenient system.^6,13−15,30,31^ We managed to identify Yfh1 residues that, when mutated, would stabilize the protein and abolish cold denaturation.^6^ We now reasoned that if we could intentionally induce cold denaturation by mutating only a few residues in an otherwise stable protein, we could claim to have understood the rules that determine cold denaturation and thus protein stability.

We chose CyaY, the bacterial orthologue of Yfh1, which shares with this protein the same fold but has a heat denaturation midpoint ca. 20 °C higher than that of Yfh1 and does not undergo cold denaturation. We found that it was sufficient to truncate CyaY C-terminally to have a protein that, under similar conditions, is strongly destabilized with a high-temperature melting point comparable to that of Yfh1.^18^ These results prove beyond any doubt the role of the C-terminus of the frataxin family in the stability of their fold as the gatekeeper of the hydrophobic core that protects the protein from unfolding. However, even though the truncated protein is strongly destabilized, it does not cold-denature at detectable temperatures. In other words, we managed to shift the stability curve toward lower temperatures, decreasing the high-temperature melting point but without substantially changing the other side of the bell-shaped stability curve.

We then introduced additional *ad hoc* mutations to induce electrostatic frustration. This was done at a site sequence-wise distant from the C-terminus but spatially close enough to the hydrophobic core and to the gate. Introduction of two newly charged groups in CyaY^103^ designed by analogy with Yfh1 and neutralization of a nearby positively charged residue (EET and EEE) destabilizes the CyaY-fold so drastically that the protein completely unfolds or misfolds in inclusion bodies. A less drastic choice (EE and ET) produces instead proteins whose stability curves are affected not only at high but also at low temperature and, in Hepes, undergo detectable cold denaturation. The effect is more pronounced in the more strained EE.

We studied the proteins’ behavior in both Hepes and phosphate, two buffers often used in biophysical studies and the same as those we had previously explored.^18^ Hepes is classified as one of Good’s buffers that are zwitterionic buffers containing aminoalkyl sulfonate.^32^ Phosphate is instead a buffer known to stabilize negatively charged proteins through different mechanisms.^33^ We found a noticeable difference between the two buffers: the mutant EET, for instance, is completely unfolded in Hepes, whereas it retains a marginal stability in phosphate. This result is consistent with our previous observations on the comparison of environmental factors on the stabilities of three frataxin orthologues^18^ and can be explained by the consideration that the larger the difference between the pI and the pH of interest, the greater the net charge on the protein.^34^ The effect is exacerbated in our case since we do not have other salts in solution. Sodium phosphate can thus be expected to stabilize the protein by shielding the net charge of the protein. However, both EE and ET, the two proteins that undergo cold denaturation at observable temperatures, have *T*_m_s higher in Hepes than in phosphate (26.5/21.7 and 36.6/31.7 for EE and ET, respectively). This is consistent with our previous observation that demonstrated a completely different mechanism for the cold and heat unfolding processes and a quite asymmetric effect of environmental factors on these processes.^23^

If we compare the unfolding profile of EE with those of Yfh1 and IscU, two natural proteins that undergo detectable cold denaturation, we find that Yfh1 still stands alone, in that the *T*_c_ of this protein is directly observable, whereas both with IscU and EE, we observe only the onset of the transition.^35^ The behavior of Yfh1 is thus more drastic, once again demonstrating the uniqueness of the features of this protein.

Some reflections are in order at this point. First, an overall protein destabilization is important to observe cold denaturation. In preliminary studies aimed at inducing cold denaturation simply by mutating wild-type CyaY to introduce the electrostatic hinge, no cold denaturation was observed (S. Gianni, personal communication). We reasoned that this is presumably because this transition may appear only in marginally stable proteins, *i.e.*, proteins with a low free energy of unfolding, which exist as an equilibrium mixture of folded and unfolded forms under “normal” conditions. We have in fact previously argued that this coexistence makes marginally stable proteins ideal tools to study even small environmental changes to which they may behave as natural sensors.^36^ Our assumption was also strongly supported by many if not all of the other examples of proteins with a detectable cold denaturation described in the literature.^9,25,37^ These considerations will need to be born in mind in future cold denaturation studies. Second, we observed that it is easy to introduce destabilization, but this should not be confused with cold denaturation. This is because it is generally assumed that thermal stability is measured by the value of *T*_m_, the temperature at which the populations of folded and unfolded forms are the same at high temperature. This is, however, true only when the corresponding stability curves are parallel. In this case, Becktel and Schellman^5^ observed that Δ*T*_m_ is proportional to ΔΔ*G*. In a more general case, this is not true: strictly speaking, *T*_m_ is not a measure of stability (Δ*G*) but just a measure of thermal resistance.^38^

In our case, we observed a concurrent down-temperature shift of *T*_m_ and a high-temperature shift of *T*_c_. Both shifts point to a decreased stability but refer to distinct unfolding mechanisms. A naïve interpretation of stability curves might imply that a decrease of *T*_m_ corresponds to a shift of the stability curve to lower temperatures and thus to a simultaneous decrease of *T*_c_ (corresponding to an increase in stability). However, a decrease of *T*_m_ at high temperature does not necessarily produce also a shift toward lower temperatures on the other side. As discussed at length in ref (31), altered thermostability can be achieved thermodynamically according to three extreme cases: a change in enthalpy (Δ*H*), a variation of curvature (Δ*C*_p_), or a shift of the maximum (*T*_s_) *versus* different temperatures.^39^ Real situations contain mixtures of the three possibilities. We have previously demonstrated that the area under the stability curve between the temperatures of cold and heat unfolding is a more reliable measure of protein stability.^13^ Accordingly, we observed here that we can destabilize the high-temperature unfolding without appreciably affecting the low-temperature point and retaining a similar *T*_s_. It is only through introducing electrostatic repulsion that we succeeded in affecting the curve not only on the *T*_s_ and on the whole area under the stability curve. This observation is fully consistent with Privalov’s theory that cold denaturation is the consequence of the opening of the hydrophobic core due to an increased interaction with the solvent, and our simulations fully support this view by showing how introduction of electrostatic strain increases the molecular motions and thus the tendency of the protein to unfold and allow solvation of the hydrophobic core.

We may also wonder how general our conclusions may be. While we cannot be sure at this stage whether destabilization with electrostatic frustration is the only route to cold denaturation, we can certainly say that we have identified another protein, bacterial and human IscU, which contains similar electrostatic frustration caused by a cluster of four negative charges and undergoes cold denaturation.^35^ Also, for this protein, we demonstrated that mutation of any of these charges is sufficient to abolish cold denaturation while not significantly affecting the high-temperature stability.

In conclusion, we have described how we succeeded in rationally inducing cold denaturation in an otherwise stable protein by the judicious choice of a few mutations able to induce overall destabilization and selective destabilization of the left side of the stability curve. We should thus conclude the conditions under which cold denaturation occurs from what we could call a “narrow tunnel” within the energy landscape of a protein in which several different criteria must be fulfilled at the same time. This, in turn, tells us how the evolution process has managed to narrowly select proteins, which, if adopting an anthropomorphic point of view, “just” manage to preserve themselves from unfolding, gaining, however, at the same time, an exquisite sensitivity to the environment, which dictates the overall balance of the forces that determine their fold under the evolutionary pressure.

## Materials and Methods

### Protein Production

Wild-type CyaY was produced as previously extensively described.^18,24^ The mutants were obtained by site-directed mutagenesis using the wild-type protein as the template. The first mutant obtained was the truncated CyaY^103^, which lacked the last three amino acids of the wild-type sequence. The other two mutants were obtained using the CyaY^103^ plasmid as the template. All mutants were cloned into a pET24(+) (Novagen, Merck, Germany) plasmid with the restriction sites NCOI and NOTI. The plasmid was a modified version with a His-tagged glutathione-*S*-transferase (GST) tag with a tobacco etch virus (TEV) cleavage site inserted between the tag and the construct of interest.^18^

All proteins were expressed in *E. coli* BL21(DE3) strain and purified as previously described.^40^ In brief, transformed cells were inoculated and grown in lysogeny broth (LB) medium with kanamycin (30 mg/mL). Expression was induced at 37 °C with isopropyl β-d-thiogalactopyranoside (IPTG) at an optical density at 600 nm (OD_600_) between 0.6 and 0.8. The cells were harvested, and the pellet was frozen to enhance the next cell lysis. The pellet was then thawed in tris–HCl 20 mM at pH 8, 150 mM NaCl, 10 mM imidazole, lysozyme, ethylenediaminetetraacetic acid (EDTA)-free DNAse, protease inhibitors, and 1 mM tris(2-carboxyethyl)phosphine (TCEP). Cell lysis was performed by sonication. Sample purification was achieved in two steps. The first step involved Ni-NTA affinity chromatography and cleavage of the His-GST tag by tobacco etch virus (TEV) protease. A further size exclusion chromatography step was then performed to separate the proteins from the His-tagged GST.

### CD Measurements

The samples (10 μM) for the CD measurements were in either 10 mM Hepes buffer at pH 7 or in 20 mM sodium phosphate (NaPi) buffer at pH 7.4. The CD measurements were carried out with a Jasco J-815 spectropolarimeter using 1 mm path length cells, a wavelength range of 190–260 nm, and 10 acquisition scans. All of the CD spectra were corrected by subtraction of the appropriate buffer spectrum. Thermal unfolding curves were obtained, as previously described,^6,18^ by monitoring the ellipticity at 222 nm over the temperature range of 3–90 °C, using 1 mm path length cells and a heating rate of 1 °C/min for all samples measured. The measurements were repeated at least three times using different protein batches. For some samples, the acquisition repeated using a heating rate of 0.2 °C/min over the temperature range of −1–10 °C and a time constant of 8 s. The temperature was monitored with a cell holder thermostated by a PTC-514 Peltier system.

### Calculation of the Thermodynamic Parameters and the Stability Curves

We derived the thermodynamic parameters from CD data and converted them into a stability curve. In brief, the CD signal at 222 nm (*S*) is proportional to the fraction of the folded protein (*f*_U_) and it can be estimated at each temperature using the equation
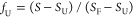
where *S* is the measured signal, *S*_U_ is the signal of the unfolded state, and *S*_F_ is the signal of the folded one. As previously discussed, the heat capacity difference between the folded and unfolded forms, Δ*C*_p_, was calculated by assuming a contribution of 14 cal/(mol K) for each residue^28^ (109 for CyaY and 106 for the truncated constructs, including three additional N-terminal amino acids deriving from the TEV cleaving site) and it is assumed to be independent of temperature. The thermodynamic parameters *T*_m_ and Δ*H*°_m_ were derived by nonlinear least-squares fitting using the Levenberg–Marquardt algorithm with the following equation
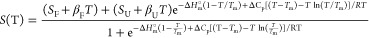
where β_F_ (β_U_) and *S*_F_ (*S*_U_) are the slopes and intercepts of the pre (and post)transition slopes.

Δ*S*°_m_ was obtained from the Gibbs–Helmholtz equation at *T* = *T*_m_
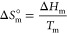
The stability curve of the protein is a plot of the difference in free energy between the folded and unfolded species Δ*G*°(*T*), as calculated from the modified Gibbs–Helmholtz equation
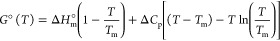


### MD Simulations

The wild-type structure of the CyaY protein was downloaded from the Protein Data Bank (code 1soy). Deletion of the three C-terminal amino acids produced the starting structure of CyaY^103^. The mutations to obtain the structures of ET, EE, and EET were generated with the “pdb4amber” module of AmberTools16 suite. All systems were studied with independent replicas of all-atom simulations and were partially neutralized (Table 2). All simulations were carried out with the Amber16 suite, using the force field FF14SB and TIP3P as the water molecule model.^41^ The simulation boxes were octahedral, with dimensions chosen to ensure 11 Å between all protein atoms and the box edges. The wild-type protein and the CyaY^103^ mutant were subjected to an unrestrained minimization consisting of 1000 steps of steepest descent followed by 1000 steps of conjugate gradient minimization. The minimized systems were then equilibrated at 300 K for 10 ns using Langevin coupling, with γ equal to 1 ps^–1^.^42^ After this step, the relaxed systems were simulated in the *NPT* ensemble at 1 atm using the Berendsen coupling algorithm (Table 2).^43^ The full particle-mesh Ewald method was used for the electrostatics.^43^ The SHAKE algorithm was used to constrain all covalent bonds involving hydrogen atoms.^44^ A 2 fs time step and a 10 Å cutoff were used for truncation of the van der Waals nonbonded interactions. Each trajectory had a different simulation time, ranging from 500 to 1000 ns, but the same simulation temperature fixed at 300 K (Table 2). Two cycles of minimization were used for ET, EE, and EET. The first step was carried out by constraining the positions of all of the Cα atoms and the second by releasing all of the constraints. We followed this procedure to release the steric clashes produced by the two mutations. After these steps, the simulation procedures were the same as those used for the wild-type and CyaY^103^. All of the structural and energetic analyses were carried out using GROMACS 467 and Amber Suite analysis tools.^45,46^ The frame frequency for all of the analysis was 25 ps, so 2000 and 4000 frames for 500 and 1000 ns, respectively.

**Table 2:** Summary of the Parameters Assumed in the Different MD Trajectories

system	replica	no. of atoms	num. of water mol.	counterions	simulation length (ns)
CyaY	1	23 754	7532	14 Na+	500
	2			8Cl^–^ 8K+	500
	3				500
CyaY^103^	1	23 868	7411	15 Na+	1000
	2			8Cl^–^ 8 K+	
ET	1	23 779	7383	17 Na+	1000
	2			8Cl^–^ 8K+	1000
	3				1000
EE	1	23 784	7384	18 Na+	1000
	2			8Cl^–^ 8K+	1000
	3				500
EET	1	23 790	7386	18 Na+	1000
	2			8Cl^–^ 8K+	1000
	3				1000

## Supporting Information Available

Comparison of the temperature dependence of the CD intensities at 222 nm for wild-type CyaY, CyaY^103^, EE, ET, and EET (PDF)
