# Supplementary material for: Recipes for Inducing Cold Denaturation in an Otherwise Stable Protein
Source: J Am Chem Soc. 2022 Apr 15;144(16):7198–207. doi: 10.1021/jacs.1c13355 (PMC9052743; doi:10.1021/jacs.1c13355)

**Supplementary materials**

# Recipes for inducing cold denaturation in an otherwise stable protein


Angela Bitonti[1,2#], Rita Puglisi[3#], Massimiliano Meli[4], Stephen R. Martin[5], Giorgio Colombo[6],

Piero Andrea Temussi[3*], Annalisa Pastore[3,7*]

[1]Department of Molecular Medicine, University of Pavia, Pavia, Italy

[3]UK Dementia Research Institute at the Maurice Wohl Institute of King's College London, London, SE5 9RT, United Kingdom

[4]Istituto di Scienze e Tecnologie Chimiche "Giulio Natta" - SCITEC, CNR, Via Mario Bianco 9, 20131 Milano (Italy)

[5]Structural Biology Technology Platform, |The Francis Crick Institute, 1 Midland Rd, London NW1 1AT, United Kingdom

[6]Department of Chemistry, University of Pavia, Pavia, Italy

Present addresses:

[2]Scuola Normale Superiore, Pisa, Italy

[7]European Synchrotron Radiation Facility, 71 Ave. des Martyrs, 38000 Grenoble, France

*To whom correspondence may be addressed

annalisa.passtore@crick.ac.uk, temussi@uninna.it

[#]The two authors have equally contributed

**Figure S1.** Comparison of the temperature dependence of the CD intensities at 222 nm for wild-type CyaY, CyaY$^{103}$, EE, ET and EET.

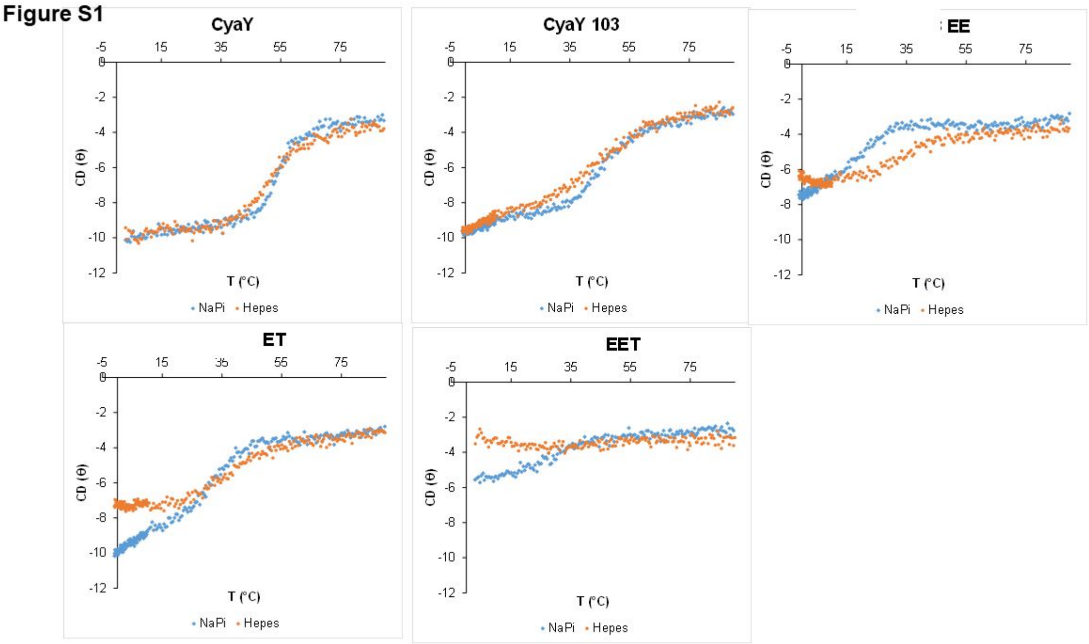

Supplement: Supplementary file 1 — ja1c13355_si_001.pdf [file ja1c13355_si_001.pdf]
